# Applications of Essential Oils as Antibacterial Agents in Minimally Processed Fruits and Vegetables—A Review

**DOI:** 10.3390/microorganisms10040760

**Published:** 2022-03-31

**Authors:** Maria Isabel S. Santos, Cátia Marques, Joana Mota, Laurentina Pedroso, Ana Lima

**Affiliations:** 1 Faculty of Veterinary Medicine, Lusófona University, 1749-024 Lisbon, Portugal; catia.marques@ulusofona.pt (C.M.); p6494@ulusofona.pt (J.M.); laurentina.pedroso@ulusofona.pt (L.P.); 2 Linking Landscape, Environment, Agriculture and Food (LEAF), Instituto Superior de Agronomia, University of Lisbon, 1349-017 Lisbon, Portugal; 3 Centre for Interdisciplinary Research in Animal Health (CIISA), Faculty of Veterinary Medicine, University of Lisbon, 1300-477 Lisbon, Portugal

**Keywords:** essential oils, minimally processed foods, foodborne pathogens, antibacterial mechanisms, minimal inhibitory concentrations, minimal bactericidal concentrations

## Abstract

Microbial foodborne diseases are a major health concern. In this regard, one of the major risk factors is related to consumer preferences for “ready-to-eat” or minimally processed (MP) fruits and vegetables. Essential oil (EO) is a viable alternative used to reduce pathogenic bacteria and increase the shelf-life of MP foods, due to the health risks associated with food chlorine. Indeed, there has been increased interest in using EO in fresh produce. However, more information about EO applications in MP foods is necessary. For instance, although in vitro tests have defined EO as a valuable antimicrobial agent, its practical use in MP foods can be hampered by unrealistic concentrations, as most studies focus on growth reductions instead of bactericidal activity, which, in the case of MP foods, is of utmost importance. The present review focuses on the effects of EO in MP food pathogens, including the more realistic applications. Overall, due to this type of information, EO could be better regarded as an “added value” to the food industry.

## 1. Introduction

### 1.1. Minimally Processed Fruits and Vegetables

**Figure 1:**
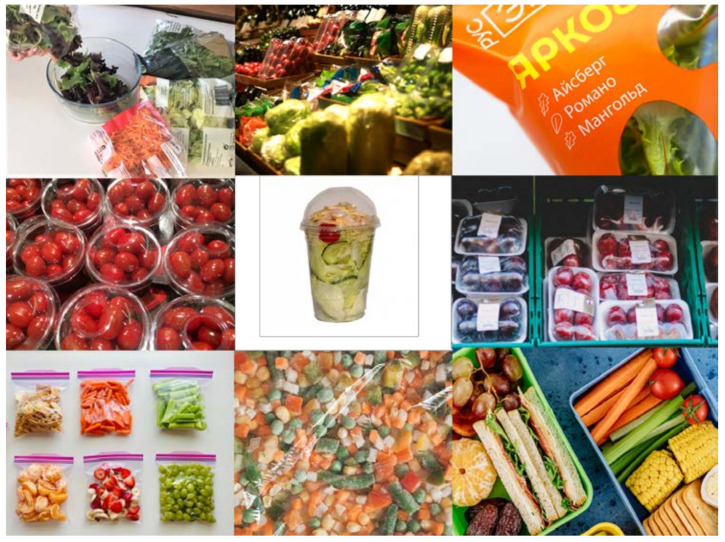
Examples of minimally processed (ready-to-eat) fruits and vegetables.

The fast pace of modern life has led to a shortage of time, particularly regarding meal preparations; there is an increase in consumer preferences for food that is healthy, fast, and easy to prepare [1,2,3,4]. The food industry—in effort to meet consumer demands—is continuously developing a wide range of ready-to-eat, fresh-cut, refrigerated foods with prolonged shelf-lives [1]. Preservation techniques, such as refrigeration, moderate heating, specific packaging, and antimicrobial disinfectants are usually applied to maintain a product’s freshness. Ready-to-eat fresh foods, with minimal alterations and without strong preservatives are referred to as minimally processed (MP) foods [1,2,4]. These new MP foods are marketed and packaged in a ready-to-eat state for ease and convenience, and they comprise a wide range of products, such as fresh cut vegetables, meat, and fish [4,5]. MP foods have emerged in response to a new market tendency, i.e., a concomitant increasing demand for efficient preservation techniques that lack the need for chemical preservatives [3]. MP vegetables/fruits are a particular branch in the MP food industry; this branch has gained much interest from consumers since MP vegetables/fruits are considered healthier than processed food products. Minimally processed fruits and vegetables (MPFVs) include any fresh vegetable or fruit that has been minimally altered (usually cut, peeled, shredded, and washed) and packaged, in a ready-to-use state, whilst remaining fresh [4,6,7,8] (Figure 1).

These types of products simplify everyday life, allowing for the preparation of healthy, enjoyable, and diversified meals, in a time-saving fashion, with reduced food waste. In the United States (US), MPFV sales grow by approximately USD 15 billion per year and represent 15% of sales in all plant products [9,10]. The best-selling product is a ready-to-eat salad, in which sales increased from USD 2.7 to 3.2 billion between 2001 and 2003. In Europe, consumption varies widely among countries, with the United Kingdom (UK) being the largest consumer, having exceeded 120,000 tons of sales in 2004 [9,10].

Nonetheless, MP foods are not sterile. As vegetables are raw and of agricultural origin, MPFVs contain microorganisms (often pathogenic) [11,12,13,14,15]. It is therefore not surprising that some of the most nutritionally recommended foods are also those with the greatest food preservation and safety challenges. Indeed, fruits and vegetables are often incriminated in foodborne diseases worldwide. In recent decades, foodborne outbreaks associated with raw fruit and vegetable consumption have increased. This has led to researchers and health authorities (in food safety areas) analyzing the microbial contamination of fresh produce [16,17,18,19,20]. There is growing concern about the potential risks of microbiological proliferation, owing to the high-levels of manipulation that these types of products are subject to and the increase in MPFV consumption worldwide. Vegetables may become contaminated in the pre-harvest stage (e.g., as a plant in the field or during harvesting) and in the post-harvest phase (e.g., during transportation, processing, and packaging) [14,21,22,23]. Thus, the microbial quality and safety of MPFVs is a serious concern.

Over the years, extensive studies have been carried out on the antimicrobial activities of essential oil (EO) and its application in food systems. The use of EOs—specifically in MPFVs—has been garnering more attention of late, but there are a lack of consolidated appraisals on this issue. Most studies focus on in vitro testing; few show applications in realistic scenarios. Therefore, the present review focuses on the effect of EO in MP food pathogens, focusing on their more realistic applications, particularly on promising innovative solutions for their safe usage. Overall, due to this type of information, EO could become an “added value” to the food industry.

### 1.2. Major Pathogens Related to Foodborne Diseases in MP Foods

In the last three decades, the epidemiology of foodborne infectious diseases has undergone a radical change; vegetal products have “arisen” as new vehicles of microorganisms [12]. There have been numerous outbreaks, as shown in the scientific literature, describing situations that have resulted in the death of hundreds of people [20,21,22,23,24]. *Salmonella* spp., *Escherichia coli* O157:H7, and *Listeria monocytogenes* are the pathogenic microorganisms that cause the most concerns in outbreaks of this nature [14,18,25]. Several of these outbreaks have led to widespread public health concerns. For example, between May and July 2011, a major outbreak occurred as result of the high number of cases and the difficulties in detecting the source of the infection. The outbreak occurred in Germany; out of a total of 3816 cases, 845 patients developed hemolytic uremic syndrome (HUS) and 54 died. Most of the patients (88%) who developed HUS were adults, contrary to what usually occurs in VTEC strain infections. Likewise, the female gender (aged between 30 and 34 years) was the most affected (68% of cases with HUS and 58% of gastroenteritis). The epidemic strain of this outbreak was an *E. coli* O104:H4 enteroaggregative that acquired the Shiga toxin 2 (Stx2a) converting bacteriophage. This outbreak disseminated worldwide, with reports in 15 European countries and in the USA. In France, eight cases occurred in people who had been present at a community event, and the isolated strain had a genetic profile compatible with the epidemic strain from Germany. Given that it was a common event, it was possible to identify the suspected food as fenugreek sprouts imported from Egypt in 2009 [26,27,28,29]. According to data from the USA, fruits and vegetables account for an estimated 46% of foodborne illnesses, most of which are caused by norovirus, *Salmonella* spp., and *E. coli* O157:H7, with leafy vegetables being the most frequent vehicle. Vegetables are responsible for 2.2 million foodborne illness cases per year (22%), corresponding to the food product responsible for the largest number of patients. It is estimated that 24,000 people (41%) are hospitalized annually due to the consumption of products of plant origin, of which, 38% are attributed to fruits and vegetables and 16% to leafy vegetables, just behind dairy products, which occupy first place (in terms of hospitalizations). Regarding the number of deaths—fruit and vegetable consumption is related to 333 foodborne illnesses per year (23%), far below the 43% from animal product consumption (terrestrial). In summary, leafy vegetables account for the largest number of patients with foodborne diseases (22%), being the second cause of hospitalization (14%) and the fifth most frequent cause of death (6%) [19].

### 1.3. Current Decontamination Methodologies for MP Foods and Related Problems

#### Current Decontamination Methodologies for MP Foods and Related Problems

Washing and disinfecting plant products are only moderately effective, as they are by no means efficient when pathogenic microorganisms are internally located. Microorganisms can penetrate plant tissues, both in the pre-harvest phase by internalization, or in the post-harvest phase by infiltration, which makes its elimination much more complex. Once bound, microorganisms can be incorporated into biofilms, which increases their ability to survive in plant tissues [30]. In short, pathogenic microorganism internalization can occur at any stage of the plant life cycle (seed, germination, mature plant, flower, fruit), moving on to the next phase [31,32]. Thus, even when disinfected, these microorganisms can be out of reach in irregular surfaces or in biofilms. Similarly, injuries caused by harvesting and transport can provide protective places where microorganisms can survive and grow, unharmed [6].

Currently, the most commonly used disinfection methods are chlorine-based [32,33], with chlorinated water being the usual selection used to disinfect MPFV due to its low cost and simplicity of use [32,33]. The effectiveness of decontamination, per se, is measured by the reduction of microorganisms obtained and, more importantly, by the ability to maintain this reduction over the product’s shelf-life. However, active chlorine is not very effective since its disinfecting power is short-lived and the surviving bacterial populations can actually multiply faster than the corresponding populations in non-disinfected products [33,34]. Furthermore, chlorine can be harmful due to the formation of toxic derivatives, such as trihalomethane and chloramine. Hence, there are health concerns associated with its use, which has led to restrictions on its use in several European countries, namely the Netherlands, Sweden, Germany, Swiss, Denmark, and Belgium [22,24,33,35,36,37,38].

Other methodologies can include the use of chlorine dioxide [4,39], organic acids [4,11,40,41,42,43], hydrogen peroxide [4,44,45,46], electrolyzed water [4,47], ozonated water [4,48,49,50,51,52], or calcium-based solutions [4,53]. It was found that, generally, these methods are easy to apply and have strong bactericidal effects. However, most present disadvantages in their use. For example, the use of chlorine dioxide has been shown to be effective in reducing bacterial populations, but it ultimately affects some organoleptic characteristics. The drastic reduction of the native microbial population is another factor to consider, i.e., decreasing competition for space and nutrients may lead to a subsequent increase in the development of pathogenic microorganisms [24,35]. Other physical treatments that have been developed in recent years include ionizing radiation [4,9,18], ultraviolet [4,54], and infrared or modified atmosphere [4,35]. These methods may be bacteriostatic or bactericidal, and they have shown high efficiency in the inhibition of microbial contaminations [35]; however, they present technological problems that limit their usefulness. For example, the irradiation process cannot be used in isolation as a step in continuous washing [55].

### 1.4. Natural Alternatives for Decontamination of MP Fruits and Vegetables

Consumers consider the use of natural antibacterial compounds as a promising alternative to chemical disinfectants, not only in the context of food safety, but also as an alternative to chemical antibacterial agents (overall) [18,56,57,58,59,60]. Several studies have been carried out in this area. Most of these have the goal of eliminating both the pathogens and the microorganisms responsible for vegetable spoilage [55,61]. The main sources of these natural antibacterial compounds are plants (e.g., essential oils), microorganisms (e.g., lactic acid bacteria through the production of lactic acid and antimicrobial polypeptides), and animals (e.g., lysozyme) [60,62]. Since these natural products and their components are accepted as safe to consume (generally recognized as safe—GRAS), concerns surrounding their safety of use in MP foods are minimal. In recent decades, studies have focused on several natural compounds that have potential in food disinfection. Some examples are acetic acid, ascorbic acid, lactic acid, essential oils, and cheese whey, among others [60,62]. Albeit, few disinfectants proposed in scientific studies have actually reached the market. Indeed, the need for more practical and realistic studies and approaches is adamant to surpass this challenge.

## 2. Essential Oils as Alternative Food Disinfectants

Since ancient times, the antimicrobial properties of plants and spices have been exploited as food preservatives [63,64,65,66,67]; scientific interest in this area has recently re-emerged [68]. In recent decades, essential oils (EOs) from aromatic and medicinal plants have been used as novel alternatives to common food antibacterial agents, as they are natural products, inherently well tolerated, and present fewer side effects when compared to other food preservatives or disinfectants. 

EOs the result of plant secondary metabolites; they are known to present intense odors, being extremely volatile and hydrophobic [69]. They are produced by specialized excretory structures and can be found in several parts of these plants, namely leaves, fruits, flowers, buds, seeds, branches, and roots, and their compositions may vary according to the location [70].

In nature, these metabolites have two distinct functions: (1) they protect plants against pests or infections through their insecticidal, antibacterial, and antifungal actions; (2) they attract certain insects, so that they remove pollen from the plant, facilitating pollination [71]. The amount and composition may vary, both genetically and physiologically, as well as due to external factors, such as growing conditions, harvesting, post-harvest conditions, and environmental factors, among others [69,72].

### 2.1. Composition of Essential Oils

EOs are volatile, natural, complex compounds formed by aromatic plants as secondary metabolites; they are characterized as having strong odors [71]. In nature, EOs play an important role in the protection of plants through their antibacterial, antiviral, antifungal, and insecticides actions, as well as against herbivores by reducing their appetite for such plants. EOs may attract some insects, to favor the dispersion of pollen and seeds, or repel others that are undesirable [71]. EO chemical compositions can widely differ, according to several factors, such as the soil composition, the organ of the plant from which it is extracted, the time of the year it is harvested, the plant and organ age [71,73], and the extraction method used [63]. The different EO compositions result in different responses in their antimicrobial activities, even when they are tested under the same conditions. Thus, obtaining/extracting in a standardized manner is important in order to obtain a constant composition of EO [63,71].

EOs are complex natural mixtures that could contain approximately 20–60 components at quite different concentrations. They are characterized by two or three major components at fairly high concentrations (20–70%) in combination with other components that are only present in trace amounts [63,71,74]. Generally, it is the component in the greatest concentration (major constituent) that confers the biological activity to the EO; however, this activity is often the result from the synergy between several components [63,74,75]. In a study carried out in the control of *Botrytis cinerea* using several EOs, the authors verified that, in most cases, those with the highest concentrations of the major constituents had higher fungicidal activities [76].

Table 1 shows the major components of some of the most known EOs used in foods. These active compounds have different chemical groups, composed of alcohols, esters, aldehydes, ketones, phenols, and phenolic ethers, with terpene compounds being the most abundant [77]. The components include two groups of distinct biosynthetic origins [63,71]. The main group is composed of terpenes and terpenoids and the other of aromatic and aliphatic constituents, all characterized by low molecular weight [71]. Terpenes form structurally and functionally different classes. They are made from combinations of several 5-carbon-base (C5) units called isoprene [74] and they have been extensively reviewed [74]. The main terpenes in EO are monoterpenes (C10) and sesquiterpenes (C15), but monoterpenes are the most representative molecules, constituting 90% of essential oils and allowing for a large variety of structures [71], although they usually do not represent a group of constituents with high inherent antimicrobial activity [74]. Hemiterpenes (C5), diterpenes (C20), triterpenes (C30), and tetraterpenes (C40) also exist [74]. Examples of plants containing these compounds are angelica, bergamot, caraway, celery, citronella, coriander, eucalyptus, geranium, juniper, lavender, lemon, lemongrass, mandarin, mint, orange, peppermint, petitgrain, pine, rosemary, sage, and thyme [71].

Terpenoids are terpenes that undergo biochemical modifications via enzymes that add oxygen molecules and move (or remove) methyl groups [71,74,77]. Terpenoids can be subdivided into alcohols, esters, aldehydes, ketones, ethers, phenols, and epoxides. Examples of terpenoids in EOs with food applications are: thymol, carvacrol, linalool, citronellal, piperitone, menthol, and eugenol (Table 1). The antimicrobial activities of most terpenoids are linked to their functional groups; the hydroxyl group of phenolic terpenoids is recognized as the most important for antimicrobial activity [74].

Besides terpenes and terpenoids, aromatic compounds occur less frequently, but are also noteworthy. They are derived from phenylpropane and include cinnamaldehyde, chavicol, eugenol, myristicin, and safrole, among others [74,77]. The main plant families for these compounds are *Apiaceae*, *Lamiaceae*, *Myrtaceae*, and *Rutaceae*, which include plant species, such as anise, cinnamon, clove, fennel, nutmeg, parsley, sassafras, star anise, and tarragon, among others [74]. Sulfur-based components from plants, such as garlic and mustard oils (e.g., glucosinolates or isothiocyanate derivatives) are also secondary metabolites often found in diverse source plants for EO [74].

#### Secondary Effects Induced by EO Components

Because of the great number of constituents, essential oils can induce secondary effects to consumers, depending on their concentrations. The use of EO in foods—besides odor and taste—can induce some secondary effects in consumers, although there are restrictions on the doses used for food applications and, most of all, for food safety issues (please see Section 3). The biological effects of EOs have been extensively reviewed elsewhere [71], mostly focusing on cytotoxicity, nuclear mutagenicity, and carcinogenicity. Cytotoxicity occurs mostly due to membrane damage [78,79,80,81,82,83,84], cytoplasm coagulation [85], and overall damage to lipids and proteins [85,86,87,88,89]. Essential oil cytotoxicity in mammalian cells is caused by the induction of apoptosis and necrosis [71]. For example, eugenol, isoeugenol, methyl eugenol, and safrole induce cytotoxicity and genotoxicity in rat and mouse hepatocytes [90], and estragole also induces cytotoxicity in hamster fibroblastic V79 cells [91]. Many studies using EO or their main components have also shown that, grosso modo, most of them do not induce nuclear mutations [71]; however, there are a few exceptions, particularly in the case of some EO constituents that can act as secondary carcinogens after metabolic activation [92]. Specific EO constituents that have been shown to induce carcinogenic metabolites in rodents include safrole (from *Sassafras albidum* EO) [90,93,94], methyl eugenol (from *Laurus nobilis* and *Melaleuca Leucadendron* EO) [90], d-Limonene (from *Citrus* EO), and estragole (from *Ocimum basilicum* and *Artemisia dracunculus* EO) [93,95]. Moreover, the EO from *Salvia sclarea* and *Melaleuca quinquenervia* can induce estrogen secretion, which in turn can trigger estrogen-dependent cancers. Moreover, the EO components containing photosensitizing molecules can also cause skin erythema or cancer [96,97].

**Table 1:** Major components of some essential oils with food application.

Common Name	Scientific Name	Major Constituent	2nd Constituent	3rd Constituent	4th Constituent	5th Constituent	References
**Amaryllidaceae**							
Garlic	*Allium sativum*	Diallyl disulfide	Allyl methyl trisulfide	Diallyl trisulfide	Diallyl sulfide	Allyl methyl disulfide	[98]
Onion	*Allium cepa*	Dipropyl disulfide	Dipropyl trisulfide	Propenyl propyl disulfide	Methyl propyl trisulfide	Allyl propyl trisulfide	[99]
**Asteraceae**							
Chamomile	*Matricaria chamomilla*	Bisabolol oxide	Camphene	Sabinene	Limonene	Cineole	[100]
**Cupressaceae**							
Juniper	*Juniperus communis*	Pinene	Myrcene	Sabinene	Limonene	Caryophyllene	[101]
**Lauraceae**							
Cinnamon	*Cinnamomum zeilanicum*	Eugenol	α-Himachalene	Bicyclogermacrene	Linalool	Nerolidol	[76]
**Lamiaceae**							
Basil	*Ocimum basilicum*	Linalool	Geraniol	Eugenol	Eucalyptol	Humulene	[102]
English Lavender	*Lavandula angustifolia*	Linalool	Linalyl acetate	Geraniol	Caryophyllene	Lavandulyl acetate	
Lavender	*Lavandula hybrida*	Octyl Acetate	Linalool	Isobornyl acetate	Camphor	α-Himachalene	[76]
Lemon Balm	*Melissa officinalis*	Neral	Nerol	Geranial	Geraniol	Caryophyllene	[103]
Marjoram	*Origanum majorana*	Terpineol	Sabinene	Cymene	Terpinene	Limonene	[104]
Oregano	*Origanum vulgare*	Thymol	Terpinene	Cymene	Carvacrol	Myrcene	[104]
Peppermint	*Mentha piperita*	Menthol	Menthone	Menthyl acetate	α-Himachalene	Eucalyptol	[76]
Rosemary	*Rosmarinus officinalis*	Eucalyptol	Camphor	Pinene	Camphene	α-Terpineol	[76]
Sage	*Salvia officinalis*	Camphor	Thujone	Cineole	Camphene	Borneol	[105]
Thyme	*Thymus vulgaris*	α-Terpinene	Cymene	Thymol	Linalool	Carvacrol	[106]
**Myrtaceae**							
Eucalyptus	*Corymbia citriodora*	Citronelal	7-Octen-1-ol	Isopulegol	Fenchyl acetate	Eucalyptol	[76]
Tea Tree	*Melaleuca alternifolia*	Terpinenol	γ-Terpinene	Eucalyptol	α-Terpinene	Cymene	[76]
Clove Tree	*Syzygium aromaticum*	Eugenol	α-Humulene	δ-Cadinene	Caryophyllene oxide	Eugenyl acetate	[76]
**Piperaceae**							
Black Pepper	*Piper nigrum*	α-Pinene	β-Phellandrene	Terpinene	Cubebene	Farnesene	[76]
**Poaceae**							
Lemon grass	*Cymbopogon citratus*	Geranial	Neral	Myrcene	Geraniol	Verbenol	[76]
Citronella	*Cymbopogon nardus*	Citronelal	Geraniol	Octenol	Elemol	Citronellyl isobutyrate	[76]
Palmarosa	*Cymbopogon martini*	Geraniol	Geranyl Acetate	Linalool	β-Ocimene	α-Himachalene	[76]
**Rutaceae**							
Bergamot	*Citrus bergamia*	Linalool	Limonene	Linalyl acetate	Terpinene	Pinene	[107]
Citron	*Citrus medica* var. *sarcodactylis*	Limonene	γ-Terpinene	Terpineol	Bisabolene	Cymene	[108]
Grapefruit	*Citrus paradisi*	Limonene	Myrcene	Pinene	Sabinene	Carvone	[109]
Lemon	*Citrus lemon*	Limonene	Pinene	Linalool	Terpineol	Linalyl acetate	[110]
Orange	*Citrus sinensis* var*. dulcis*	Limonene	Myrcene	Pinene	Caproaldehyde	Sabinene	[76]
Tangerine	*Citrus nobilis* var*. tangerine*	Limonene	Linalool	Pinene	Myrcene	Terpineol	[111]
**Zingiberaceae**							
Cardamom	*Elettaria cardamomum*	Terpinyl acetate	Cineole	Sabinene	Terpineol	Limonene	[112]
Ginger	*Zingiber officinale*	Zingiberene	Citronellyl	Phellandrene	Camphene	A-Pinene	[113]

### 2.2. Antibacterial Activities of EOs in Food Safety

**Figure 2:**
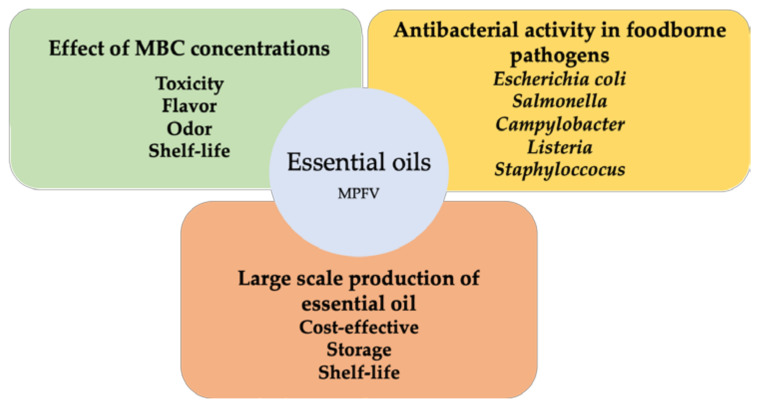
Factors affecting the practicality of essential oil antibacterial activity of minimally processed foods in the food industry.

Although the antimicrobial activities of EOs are well recognized and substantiated by many studies, their underlying antimicrobial mechanisms are still poorly understood [74]. It has been well recognized that Gram-positive bacteria is the most susceptible to EO, as opposed to Gram-negative bacteria [63,65,74,114,115,116,117,118], possibly due to their different cell wall constituents, which might hinder diffusion [63]. According to some authors [74], the antibacterial mechanisms of EO hold several targets, making it rather difficult to predict the susceptibility of a microorganism to a particular EO. Nonetheless, overall antimicrobial activity is mostly attributed to the EO’s hydrophobic nature, which allows it to effectively move across the lipid layer of the cell membranes, eventually leading to alterations in permeability and eventually disruption, culminating in the release of ions and intracellular components [119], resulting in cellular death. The overall antibacterial mechanisms encompassed by EO have been extensively reviewed elsewhere [120]. Overall, the main EO constituents (Figure 2, Table 1) are those playing the key roles in antibacterial activities, namely terpenes and other compounds, including ketones (e.g., β-myrcene, α-thujone, or geranyl acetate) and phenols (e.g., cinnamaldehyde, carvacrol, eugenol, or thymol) [121]. Carvacrol, eugenol, and thymol have been recognized as some of the major antibacterial compounds in EO [121], although many others are being reported on.

The detection of antibacterial activity in EO is of extreme importance in the food industry, to tackle the growing concerns about pathogenic and/or resistant bacteria dissemination worldwide, including via food chain transfers. Concerning the concentration range—there are several terms used in the literature to define the antimicrobial activities of EO, which are summarized in Table 2. The different definitions differ among the studies, often making it difficult to compare the results reported in various works. In the context of food safety, however, it is important to evaluate the minimum bactericidal concentration (MBC) as well as the (usually much lower) minimum inhibitory concentration (MIC) values, since the elimination of the inoculum is desirable and not only a reduction of its growth.

## 3. Challenges of the Application of EOs in MP Foods: Are They as Good as They Are Claimed to Be?

The applications of EOs in foods—due to their importance as possible alternatives as food preservatives—have been extensively reviewed [63,68,70,119,121,129,130,131]. In MP foods, in particular, the latter are much more important in food safety and industrial-scale sanitizers, where the complete elimination of foodborne pathogens from processed fruits and vegetables is required. However, despite the high number of published data about EO antibacterial activity in products such as meat, fruits, and vegetables, most studies report on MICs while only a few determine the EO MBCs [132]. A previous study by Santos et al. [132] tested and compared the MIC and MBCs of several EOs (*Origanum vulgare*, *Salvia lavandulifolia*, *Salvia officinalis*, *Salvia sclarea*, and *Rosmarinus officinalis*) as disinfectants in fresh lettuce and compared both MICs and MBCs in all tested EOs. The authors concluded that realistic antibacterial activity required the use of much higher EO concentrations than what was found in MICs, precluding its practical use. Furthermore, when testing realistic MBC concentrations, the EOs studied were also found to be active against just a small number of bacterial species (as opposed to what the in vitro MICs suggested), which further limited its usefulness as broad-range disinfectants. Furthermore, it should be noted that, above certain concentrations, EOs may no longer be viable for food use because (1) they become too odoriferous and unpalatable to taste [117] and (2) the great majority present toxicities to consumers [63,118].

Several studies have substantiated this notion. Frangos et al. [117] reported that the although the use of salt and 0.2% (*v*/*w*) oregano oil in cooked trout produced a distinct odor it was, nonetheless, well received in sensorial analysis, unlike t higher concentrations of 0.4% oregano oil (*v*/*w*) combined with salt. Mejlholm and Dalgaard [133] also concluded that for several EO, the concentrations required for extending shelf-life conveyed overly strong flavors, which limited their use.

It becomes therefore important to compare the MIC and MBC values in studies using EOs as food antibacterial agents. Table 3 presents the results of several studies in which EO MBC and MIC values were determined.

As observed, most studies report very high MBC values (over 10 μg/mL and often much more) when compared to MIC levels. Hence, although the antimicrobial activities of EOs can be well established, their practical applications in food products, particularly in MP foods, can be limited because their realistic applications would most likely produce strong and unpleasant odors, as well as undesirable changes in taste [68,74,145]. There is also further risk of toxicity for human consumption as well in such high concentrations. For example, sage EO, which is interdicted at high levels because of its high toxicity [118]. Additionally, it has been reported that, whilst many EOs may show good antimicrobial performances in vitro, they require greater concentrations to obtain similar results in food products [63].

Several other factors (apart from the limitations related to high concentrations) also challenge the use of EOs as disinfectants in MP foods. The factors affecting the practicality of EO antibacterial activity in the food industry of MPFV are depicted in Figure 2. Both physical and environmental factors can significantly interfere with the EO antibacterial activity, such as the low temperatures applied to MPFV [145]. Furthermore, the variations in EO compositions due to environmental factors [71] and the extraction methods used [63], which often lead to a lack of reproducibility [73,120].

Considering that (1) the EO constituents can often interact with food matrix components, such as fat [146,147,148], starch [149], or proteins [74,86,150]; (2) their bioactivity depends on factors such as pH [135], temperature [132,135], and the level of microbial contamination [151]; and (3) EOs--when extrapolating in vitro tests to realistic conditions—usually present lower performances [72,152], one might ask: although EOs are unequivocally good antibacterial agents, are they suitable for the MP food industry? Most works suggest that perhaps not, at least, not in the more classical context. However, several authors have suggested other approaches, such as mixing EOs with other food ingredients or other antimicrobial agents, (e.g., antibacterial peptides, such as nisin), which could facilitate the use of lower EO concentrations [153,154,155]. Nonetheless, it is important to note that many of these constraints are only observed in MPFV foods. In fact, EOs that are GRAS have been well applied to other food products, such as dairy products, sauces, desserts, and beverages [64,156], where hiding the EO odor and taste is not much of a challenge [115]. Crude EOs that are GRAS by the FDA include (amongst others) nutmeg, basil, oregano, thyme, mustard, clove, and cinnamon, amongst others [74]. Moreover, a range of EO components are used for flavoring agents in the food industry, such as thymol, eugenol, vanillin, and limonene, among others [115]. Carvacrol (having lower MIC and less toxicity) is also commonly used as a preservative and flavoring agent in food products, such as drinks and sweets [120].

## 4. Realistic Applications of EOs in MP Foods

**Figure 3:**
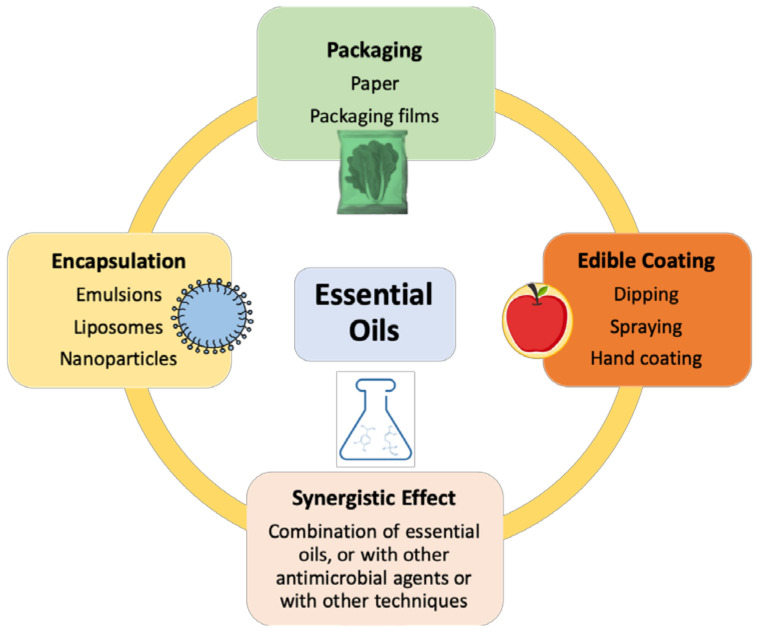
Innovative applications of EOs in MP foods.

The application of EOs in real food systems as antibacterial agents, despite its many constraints, has emerged at the lab-scale. In the last twenty years, several alternative and rather innovative EO applications have been proposed that provided effective solutions to the challenges described in this review. Most of these innovative applications allow for the use of smaller amounts of EO or avoid its contact with food products, per se. Some examples include the use of EO in packaging, coating, nanoencapsulation, and even synergistic pairing with other EO or antibacterial agents. Figure 3 summarizes the innovative applications of EO currently in use in MPFVs.

The first emerging application involves the use of EO in food packaging. Currently, a range of complementary techniques are used in MPFV packaging, such as modified atmosphere packaging (MAP) and controlled atmosphere (CA). The addition of EO in the food packaging film rather than its addition to the food product per se is considered one of the most efficient strategies used against many pathogens in MPFVs [73,120]. EO can also be encapsulated and co-polymerized into edible or biodegradable films or coatings around food products, providing its slow release to the food or to the gaseous environment of the package [68,74,157,158,159,160,161]. In some cases, the edible film or coating combines the EO with other antimicrobial agents as well [157,161,162]. Another way to optimize the use of EO is to encapsulate it into nanoemulsions. This will not only increase the volatile component’s stability, but it will also reduce interactions with the food matrix [162]. For example, Munekata et al. [146] described the use of EO against *E. coli* in fresh vegetables, often comprising washing/rinsing solutions with nanoemulsions.

Another alternative involves the combination of different EOs to obtain a synergistic effect [163]. Indeed, the combination of EOs, such as oregano, cinnamon, garlic, coriander, rosemary, sage, clove, and others, has been studied and well-reviewed [86]. Indeed, both synergistic and antagonistic effects have been reported on; this has become quite an expanding area of research with promising results [63,77,82,163,164,165,166,167,168,169]. Nonetheless, little is known about the mechanisms that rule these synergetic and antagonistic behaviors among EO components and their safety levels for consumers [164].

Overall the literature shows that these innovative EO applications are steadily revealing themselves as promising natural and effective methods used to avoid pathogenic foodborne contamination and growth in MPFVs. Although studies on antibacterial activities are scarcer than antifungal activities, there has been an undeniable increase in their use and testing. Table 4 presents the realistic and effective applications of EOs as anti-bacterial agents in MPFVs.

Despite the promising results regarding the alternative applications of EO in MPVFs, most studies fail to evaluate its impact in food quality, concerning sensorial and organoleptic qualities. Notably, the studies that do evaluate these features seem to show that the use of EO through these innovative applications does not interfere with food quality, and in some cases, can even improve the visual aspects and taste of the produce by reducing spoilage [170,171,172,173,174,179,182,184,188,189,192,194,197,201,204,205,207,208,209].

## 5. Conclusions

There are microbiological quality challenges associated with the preservation of MPFVs, which may lead to outbreaks of foodborne diseases. At present, the most widely used disinfection methods are both toxic and ineffective [34]. In this context, the use of EO has several economic, environmental, and health benefits. Thus, the use of these products in techniques involving quality preservation and food safety could signify great potential in disinfecting MPFVs. However, it is not enough to identify a good antibacterial agent, as it must also be applicable (in the food industry context). This requires a number of conditions—that are often not studied on—as a follow-up to the various published scientific studies. A good food disinfectant for MPFV should:

(1) Be effective at the indicated doses;
(2) Not be toxic, corrosive, or irritating;
(3) Be easy to prepare and apply, at a large scale;
(4) Be cost-effective;
(5) Not negatively affect the product’s organoleptic characteristics.

Few EOs, contrary to common opinion, show realistic potential as perfect disinfectants. Nevertheless, a growing body of evidence is showing that there are alternative methods to incorporate EO in MPFV food preservatives while minimizing its negative effects. Future research should therefore focus on technologically innovative applications of EO in MPFVs, using realistic EO concentrations, with knowledge-based information, aimed at practical applications in real-life scenarios. Whilst the antibacterial mechanisms of EOs render them as good alternatives to antimicrobials, even in the case of antibiotic resistance, there is still a considerable amount of work to conduct in order for their full potential to be utilized as food preservatives in MPFVs. Important aspects to consider, which are often neglected, include the use of realistic approaches, standardization of assays (e.g., in temperature, pH, and EO composition), and evaluations of the impacts of these compounds on the healthy and desirable microbiota of food products, per se.

## Figures and Tables

**Table 2:** Terms used to define the antimicrobial activities of essential oils.

Terms	Definitions	References
Minimal inhibitory concentration	Lowest concentration resulting in maintenance or reduction of inoculum viability of the tested organism.	[122]
	Lowest concentration inducing a significant decrease in inoculum viability (>90%).	[123]
	Lowest concentration inducing a complete inhibition of the tested organism, up to 48 h of incubation.	[124]
	Lowest concentration inducing visible growth reduction of the tested organism.	[77]
	Lowest concentration reducing visible growth of the tested organism	[125]
	Lowest concentration inhibiting visible growth of the tested organism over 18 to 24 h.	[126]
Minimal bactericidal concentration	Lowest concentration at which no growth is observed after subculture.	[127]
	Concentration inducing death of 99.9% or more of the initial inoculum.	[123]
	Lowest concentration that results in the death of 99.9% of the tested organism.	[125]
	Minimum concentration that induces a bactericidal effect, determined by re-culturing broth dilutions that inhibit bacterial growth (i.e., those at or above the MIC).	[126]
Bacteriostatic concentration	Lowest concentration stopping bacterial growth in broth, but cultured when broth is plated onto agar.	[128]
Bactericidal concentration	Lowest concentration stopping bacterial growth in broth; not cultured when broth is plated onto agar.	[128]

**Table 3:** Minimal inhibitory concentration (MIC) and minimal bactericidal concentration (MBC) values of essential oils against foodborne pathogens found in the literature.

Essential Oil	Microbial Strains Tested	MIC	MBC	References
*Baccharis dracunculifolia*	*Enterobacter cloacae* (clinical isolate) *Escherichia coli* ATCC 35218	6.3 mg/mL	8.4 mg/mL	[134]
	*Listeria monocytogenes* NCTC 7973 *Salmonella* Typhimurium ATCC 13311	12.7 mg/mL	16.9 mg/mL	
	*Micrococcus flavus* ATCC 10240	3.15 mg/mL	4.2 mg/mL	
	*Pseudomonas aeruginosa* ATCC 27853	1.05 mg/mL	2.1 mg/mL	
*Cinnamomum cassia*	*Listeria monocytogenes* NCTC 11994	0.5 µL/mL	0.5 µL/mL	[116]
	*Listeria monocytogenes* S0580 *Escherichia coli* O157:H7 S0575	0.3 µL/mL	0.3 µL/mL	
	*Salmonella* Typhimurium ATCC 14028 *Salmonella* Typhimurium S0584	0.25 µL/mL	1 µL/mL	
	*Klebsiella pneumoniae* ATCC 10031	2.5 mg/mL	2.5 mg/mL	[135]
	*Pseudomonas aeruginosa* ATCC 27853	5 mg/mL	5 mg/mL	
*Cinnamomum verum*	*Listeria monocytogenes* NCTC 11994 *Salmonella* Typhimurium ATCC 14028 *Escherichia coli* O157:H7 ATCC 35150	0.5 µL/mL	0.5 µL/mL	[116]
	*Escherichia coli* O157:H7 S0575 *Listeria monocytogenes* S0580	0.5 µL/mL	1 µL/mL	
*Eugenia caryophyllus*	*Listeria monocytogenes* NCTC 11994 *Listeria monocytogenes* S0580	1 µL/mL	>1.5 µL/mL	
	*Salmonella* Typhimurium ATCC 14028 *Salmonella* Typhimurium S0584	1 µL/mL	1.5 µL/mL	
	*Escherichia coli* O157:H7 S0575 *Escherichia coli* O157:H7 ATCC 35150	1 µL/mL	1 µL/mL	
*Lavandula angustifolia*	*Enterococcus faecalis* ATCC 29212 *Staphylococcus aureus* ATCC 25923	32 µL/mL	64 µL/mL	[136]
	*Escherichia coli* ATCC 25922	128 µL/mL	512 µL/mL	
*Matricaria chamomilla*	*Staphylococcus aureus* ATCC 29213 *Staphylococcus aureus* ATCC 43300 *Staphylococcus epidermidis* ATCC 12228 *Enterococcus faecalis* ATCC 51299	>4 µL/mL	>4 µL/mL	[137]
*Melaleuca alternifolia*	*Lactobacillus* spp.	1 µL/mL	2 µL/mL	[138]
	*Enterococcus faecalis* ATCC 29212	64 µL/mL	64 µL/mL	[136]
	*Escherichia coli* ATCC 25922	2 µL/mL	2 µL/mL	
	*Staphylococcus aureus* ATCC 25923	1 µL/mL	2 µL/mL	
*Mentha suaveolens*	*Salmonella* CECT 915	0.5 µL/mL	1 µL/mL	[139]
*Mentha × piperita*	*Clostridium perfringens*	10 mg/mL	10 mg/mL	[140]
*Ocimum basilicum*		5 mg/mL	5 mg/mL	
	*Staphylococcus aureus* ATCC 29213 *Staphylococcus epidermidis* ATCC 12228	0.25 µL/mL	0.25 µL/mL	[137]
	*Enterococcus faecalis* ATCC 51299	4 µL/mL	4 µL/mL	
*Pimpinella anisum*	*Clostridium perfringens*	10 mg/mL	20 mg/mL	[140]
*Origanum* sp.	*Escherichia coli* *Salmonella* Indiana *Listeria innocua* *Staphylococcus aureus*	0.9 mg/mL	1.1 mg/mL	[141]
*Origanum elongatum*	*Escherichia coli* 0157:H7	0.5 µL/mL	0.5 µL/mL	[139]
*Origanum majorana*	*Clostridium perfringens*	5 mg/mL	5 mg/mL	[140]
*Origanum vulgare*	*Salmonella* Enteritidis ATCC 13076 *Escherichia coli* ATCC 25922	320 µg/mL	320 µg/mL	[142]
	*Salmonella* Typhimurium ATCC 14028	160 µg/mL	320 µg/mL	
	*Staphylococcus aureus* ATCC 25923	640 µg/mL	>2560 µg/mL	
	Methicillin resistant *Staphylococcus aureus* ATCC 43300	320 µg/mL	1280 µg/mL	
	*Bacillus cereus* ATCC 11778	160 µg/mL	1280 µg/mL	
*Origanum vulgare* ecotype S	*Proteus mirabilis* ATCC 25933 *Proteus vulgaris* ATCC 13315	100 µg/mL	100 µg/mL	
*Origanum vulgare* ecotype SG	*Streptococcus faecalis* ATTC 29212	100 µg/mL	100 µg/mL	
*Rosmarinus officinalis*	*Salmonella* spp. (strains: 6554, 6877, 6907, 7643, 9487, 9340, 9681, 9812) ^#^	12.5 mg/mL	25 mg/mL	[143]
	*Clostridium perfringens*	10 mg/mL	10 mg/mL	[140]
	*Escherichia coli*	4.4 mg/mL	4.4 mg/mL	[139]
	*Salmonella* Indiana	8.8 mg/mL	NA	
	*Listeria innocua*	8.8 mg/mL	NA	
*Satureja montana*	*Salmonella* spp. (strains: 6554, 6877, 6907, 7215, 7466, 9487, 9681) ^#^	0.4 mg/mL	39 mg/mL	[143]
*Thymus vulgaris*	*Salmonella* Typhimurium LT2 DT104 *Salmonella* spp. (strains: 6877, 6907, 7466, 7643, 9487, 9681, 9983)	1.6 mg/mL	1.6 mg/mL	[143]
*Thymus vulgaris thymoliferum*	*Listeria monocytogenes* S0580 *Escherichia coli* O157:H7 ATCC 35150	0.25 µL/mL	0.25 µL/mL	[116]
	*Salmonella* Typhimurium (ATCC 14028; S0584) *Escherichia coli* O157:H7 S0575	0.25 µL/mL	0.5 µL/mL	
*Thymus daenensis*	*Escherichia coli*	4 mg/mL	4 mg/mL	[144]

NA: no antimicrobial activity; ^#^ *Salmonella* strains isolated from food.

**Table 4:** Overview of studies testing realistic applications of essential oils or their components as antibacterial agents in minimally processed fruits and vegetables.

Food Group	Food	Essential Oil (or Component)	Targeted Bacteria	Type of Application	References
Fruits	Table grapes	Eugenol and thymol	Natural microbiota	MAP	[170]
	Table grapes	Eugenol, thymol, and carvacrol	Natural microbiota	MAP	[171]
	Sweet cherries	Eugenol, thymol, menthol, eucalyptol	Natural microbiota	MAP	[172]
	Blueberries	Thymol	* Escherichia coli * O157:H7, *Salmonella* Typhimurium, *Listeria monocytogenes*	Washing solution	[173]
	Plums	Lemongrass	*Escherichia coli*, *Salmonella* Typhimurium	Coating	[174]
	* Avocado *	Thyme	Natural microbiota	MAP	[175]
	Pomegranate arils	*Satureja hortensis*	Natural microbiota	Dipping solution with encapsulation of EO in chitosan nanoparticles	[176]
	Fresh cut honeydew melon	Carvacrol, cinnamic acid	Natural microbiota	Dipping solution	[177]
	Fresh cut kiwi	Carvacrol, cinnamic acid	Natural microbiota	Dipping solution	[177]
	Fresh sliced apples	Hexanal, hexyl acetate, E(2)hexenal	*Salmonella enteritidis*, *Escherichia coli*, *Listeria monocytogenes*	Dipping solution	[178]
	Fresh sliced apples	Oregano, lemongrass,	Natural microflora and inoculated *Listeria innocua*	Edible coating	[179]
	* Fresh cut apples *	Citron EO, hexanal, E(2)hexenal, Citral, carvacrol	Natural microbiota *Listeria monocytogenes*, *Escherichia coli*, *Salmonella enteritidis*	Dipping solution	[180,181]
	* Apple pieces *	Lemongrass	*Escherichia coli*, endogenous microflora	Coating	[182]
	Fresh cut apples	Vanillin	*Escherichiacoli* O157:H7, *Listeria* spp.	Dipping solution	[179]
	Fresh cut apples	Eugenol and citral	* Listeria monocytogenes * and *Salmonella* Typhimurium	Edible coating	[183]
	Cut persimmon	Thyme and lemon EO	Natural microbiota	Washing solution	[184]
	Apple juice	Carvacrol, oregano oil, geraniol, eugenol, cinnamon leaf oil, citral, clove bud oil, lemongrass oil, cinnamon bark oil and lemon oil	*Escherichia coli* O157:H7	Suspensions of oils in apple juices	[185]
	Apple juice	Melissa oil, carvacrol, oregano oil, terpineol, geraniol, lemon oil, citral, lemongrass oil, cinnamon leaf oil, and linalool	*Salmonella enterica*	Suspensions of oils in apple juices	[185]
	Fruit salads	Citral Citron EO	*Salmonella* Enteritidis, *Escherichia coli*, *Listeria monocytogenes*	EO added in the syrup	[186]
Vegetables	Romaine lettuce	Thyme	* Escherichia coli * O157:H7	EO added to washing water	[49]
	Romaine lettuce	Thymol	* Escherichia coli * O157:H7, *Salmonella* Typhimurium, *Listeria monocytogenes*	Washing solution	[172]
	Iceberg lettuce	Basil methyl chavicol	Natural microbiota	Washing solution	[63]
	* Iceberg lettuce *	Oregano and rosemary	*Listeria monocytogenes*, *Yersinia enterocolitica*, and *Aeromonas hydrophila*	Dipping solution	[187]
	* Lamb’s lettuce *	Oregano and thyme EO	Natural microbiota *Listeria monocytogenes*, *Escherichia coli*	Dipping solution	[188]
	* Lamb’s lettuce *	Oregano and thyme EO	*Listeria monocytogenes*, *Salmonella enteritidis*, *Escherichia coli*, *Staphylococcus aureus*	Washing solution	[189]
	* Lettuce *	Oregano EO	* Salmonella * Typhimurium	Washing solution	[190]
	Fresh lettuce	Oregano oil	*Escherichia coli* , *Listeria monocytogenes*, *Salmonella* Typhimurium	Washing in nanoemulsions	[191]
	Fresh-cut lettuce	* Origanum majorana * EO	Natural microbiota	Dipping solutions in combination with ascorbic acid and chitosan	[192]
	Rucola leaves	Lemon oil	Natural microbiota	Coating	[193]
	Green beans	Tea tree and peppermint EO	Natural microbiota	Dipping solution	[194]
	Green beans	Carvacrol	* Escherichia coli* , *Salmonella* Typhimurium	MAP	[195]
	Green beans	Mandarin oil	* Listeria innocua *	Combined coating and γ-irradiation treatment	[196]
	Carrots	Thyme	* Escherichia coli * O157:H7	EO added to washing water	[49]
	Fresh Baby carrot	Pullulan–caraway	* Salmonella * Enteritidis, *Staphylococcus aureus*	Coating with pullulan films containing EO	[197]
	Zucchini	Carvacrol	* Escherichia * * coli *	Washing with nanoemulsions	[198]
	Spinach leaves	Carvacrol/ Eugenol	*Escherichia coli* , *Salmonella enterica*	Washing with nanoemulsions	[199]
	Cucumber slices	Carvacrol	* Escherichia coli *	Coating and combined with pulsed light	[200]
	Fresh shredded cabbage	Mint or thyme	* Listeria monocytogenes *	MAP with EO imbibed in chitosan film	[201]
	Broccoli florets	Mandarin	* Listeria monocytogenes *	Coating	[202]
	* Four season salad *	Oregano EO and citral	Natural microbiota	MAP	[203]
	Eggplant salad	Oregano oil	* Escherichia coli * O157:H7	EO mixed added directly to the food product	[204]
	Fresh leafy vegetables with red beet	Spanish origanum, Spanish marjoram, and coriander	* Listeria monocytogenes *	Dipping solution	[205]
	Fresh-cut vegetables	Thyme, oregano, and rosemary	*Listeria monocytogenes*	MAP + shredded fresh herbs (thyme, oregano and rosemary)	[206]
	Fresh-cut mixed celery, leek and butternut squash	Tea tree	* Escherichia coli * O157:H7	Combination of bioactive agents (tea tree EO, propolis extract, and gallic acid) and storage temperature	[207]
	Lettuce, carrot and red cabbage	Oregano and citral	* Escherichia coli* , *Salmonella enterica*, *Listeria monocytogenes* and natural microflora	MAP	[208]
	Broccoli and radish sprouts	Carvacrol	*Salmonella* Enteritidis and *Escherichia coli* O157:H7	Nanoemulsified carvacrol washing solution	[209]

## Data Availability

Not applicable.
